# Aristolochic acid nephropathy: A scientometric analysis of literature published from 1971 to 2019

**DOI:** 10.1097/MD.0000000000026510

**Published:** 2021-07-09

**Authors:** Hongjian Ji, Jingyin Hu, Guozhe Zhang, Jianxiang Song, Xiaohua Zhou, Dean Guo

**Affiliations:** a School of Chinese Materia Medica, Nanjing University of Chinese Medicine, Xianlin Road #138, Nanjing; b Shanghai Institute of Materia Medica, Chinese Academy of Sciences, Shanghai; c Department of Pharmacy, Department of Nephrology, The Sixth Affiliated Hospital of Nantong, Yancheng; d College of Traditional Chinese Medicine, Shanghai University of Chinese Medicine, Shanghai; e Department of Pharmacy, Jiangsu Vocational College of Medicine, Yancheng, China.

**Keywords:** aristolochic acid nephropathy, Balkan endemic nephropathy, scientometrics

## Abstract

**Background:**

Aristolochic acid nephropathy (AAN) is a type of drug-induced nephropathy that may result in acute kidney injury and is associated with a potentially progressive course of kidney fibrosis and upper tract urothelial carcinoma. Aristolochic acids (AAs) are a group of toxins commonly present in plants of the genera *Aristolochia* and *Asarum*, which are found worldwide. AAN still occurs in Asian and Balkan regions. The progressive lesions and mutational events initiated by AAs are irreversible, and no effective therapeutic regimen for AAN has been established. Furthermore, more people are at risk of this disease due to casual exposure to AAs. This study performed a scientometric analysis of global research literature focusing on AAN.

**Methods:**

The Web of Science database was searched to identify all publications pertaining to “aristolochic acid nephropathy” or “Balkan endemic nephropathy” using these terms as key words to search the literature from 1971 to 2019. The collected data included the document type, author, journal, publication year, citation reports, and country of publication, and were analyzed using the VOSviewer software.

**Results:**

A total of 1251 records were initially obtained. Publication types, including “meeting abstract,” “letter,” “editorial material,” and “proceedings paper” were excluded, which left 1083 publications comprising 923 articles and 160 reviews. English was the predominant language of the publications. China had the most number of articles published with 217 (20.0%), followed by the USA with 186 articles (17.2%), and Germany with 138 articles (12.7%). *Kidney International*, *Food and Chemical Toxicology*, and *Toxins* were the 3 most active journals in publishing articles related to AAN. The total number of citations received by all publications was 39,970, with an average of 36.91 citations per article (range: 0–1769). The literature mainly focused on apoptosis, oxidative stress, and inflammation in AAN.

**Conclusion:**

This study indicated that AAN is a significant topic in nephrology research, as shown by the large number of publications. The literature has mainly focused on the mechanisms of AA-induced nephropathy.

## 1 Introduction

Aristolochic acid nephropathy (AAN), which results in progressive renal interstitial fibrosis frequently associated with urothelial malignancies, was initially reported in Belgian patients after ingestion of aristolochic acids (AAs).^[1,2]^ Although botanicals that are known or suspected to contain AAs are no longer permitted in many countries, the incidence of AAN is probably much higher than initially thought, particularly in Asia.^[3]^ In Asian countries, especially China, traditional medicines for self-medication or agricultural products sold by networks have become a trend. Owing to a similar pronunciation, AA-containing herbs are always misused for “*mutong*” or “*fangchi*,” which belong to *Akebia Decne* genus and *Menispermaceae* family, respectively.^[4]^ Balkan endemic nephropathy is highly prevalent; every year, about 25,000 people suffer from this disease and nearly 100,000 people are at risk in the Balkan region, especially in the endemic farming villages.^[5–7]^ Recent investigations have shown that this is caused by flour being inadvertently contaminated with traces of AAs.^[8,9]^

Scientometrics or bibliometrics are methodological approaches aimed at providing quantitative and qualitative analysis of existing scientific literature on a large variety of topics. These have included themes such as nonalcoholic steatohepatitis,^[10]^ curcumin,^[11]^ Parkinson's diseases,^[12]^ and Qigong.^[13]^

To our knowledge, no scientometric studies have been carried out at the global level to exclusively assess AAN. The aim of this study was to provide accurate data on worldwide research productivity and publication trends in the field of AAN using a scientometric approach.

## 2 Methods

We searched the Web of Science database for the period from1971 to 2019 to identify all articles pertaining to “aristolochic acid nephropathy” or “Balkan endemic nephropathy” using these terms as the keywords in the search. The Web of Science database was selected for this scientometric study as it provides detailed information about the ranking of authors, country affiliations, journals, and citations. Most scientometric indicators are presented in a table or figure format.

### 2.1 Statistical methods

We included studies published in all languages. The collected data included the number of articles published annually, document type, Hirschindex (h-index), authorship, source journal, publication year, number of citations, and country of publication. The ranking of authors, country affiliations, journals, and citations were analyzed by Microsoft Excel and are presented in Tables 1–3. The full records and cited references of these publications were loaded into VOSviewer for further scientometric analyses. The co-occurrence and cluster analysis of keywords were also carried out by using VOSviewer software.

**Table 1:** Top 10 most productive journals.

Rank	Journal	N (%)	Impact factor	H-index	Average citations per item
1	Kidney International	44 (4.1%)	8.945	23	46.14
2	Food and Chemical Toxicology	30 (2.8%)	4.531	18	31.77
3	Toxins	24 (2.2%)	4.679	14	44.96
4	Nephrology Dialysis Transplantation	23 (2.1%)	3.531	16	31.91
5	Renal Failure	21 (1.9%)	2.089	9	9.05
6	Archives of Toxicology	19 (1.8%)	1.985	15	45.74
7	Toxicology	17 (1.6%)	5.059	15	43.35
8	PLOS One	16 (1.5%)	2.740	10	21.13
9	American Journal of Kidney Diseases	14 (1.3%)	4.099	13	85.43
10	Chemical Research in Toxicology	14 (1.3%)	6.618	9	32.86

**Table 2:** Top 10 publications selected by citations.

Rank	Authors	PubMed ID	Country	Year	Journal	Cited by
1	Jha et al^[16]^	23727169	India	2013	Lancet	1687
2	Vanherweghem et al^[17]^	8094166	Belgium	1993	Lancet	756
3	Nortier et al^[18]^	10841870	Belgium	2000	New England Journal of Medicine	683
4	Pfohl-Leszkowicz et al^[19]^	17195275	France	2007	Molecular Nutrition & Food Research	628
5	Peraica et al^[20]^	10534900	Croatia	1999	Bulletin of the World Health Organization	439
6	Debelle et al^[21]^	18418355	Belgium	2008	Kidney International	416
7	Grollman et al^[22]^	17620607	USA	2007	Proceedings of the National Academy of Sciences of the United States of America	389
8	Arlt et al^[23]^	12110620	England	2002	Mutagenesis	327
9	Marquardt et al^[24]^	1474034	Canada	1992	Journal of Animal Science	319
10	Finkelman et al^[25]^	Not reported	USA	2002	International Journal of Coal Geology	284

**Table 3:** The top 20 recurring terms from keywords.

Keyword	Occurrence (% of 1083 publications)
Balkan endemic nephropathy	410 (37.9%)
Aristolochic acid	310 (28.6%)
Chinese herbs nephropathy	203 (18.7%)
Nephropathy	194 (17.9%)
Ochratoxin A	172 (15.9%)
Aristolochic acid nephropathy	131 (12.1%)
Toxicity	110 (10.1%)
DNAadducts	96 (8.9%)
Kidney	95 (8.8%)
Nephrotoxicity	88 (8.1%)
Rats	86 (7.9%)
Etiology	85 (7.8%)
Chinese herbs	81 (7.5%)
Cancer	80 (7.4%)
Urothelial carcinoma	77 (7.1%)
Exposure	75 (6.9%)
Apoptosis	73 (6.7%)
Urothelial cancer	70 (6.4%)
DNA adduct formation	60 (5.5%)
Reductive activation	57 (5.3%)

### 2.2 Constructing bibliometric networks using VOSviewer software

The VOSviewer software (v.1.6.16) extracted and analyzed the semantic contents of the authors and keywords of the publications, related them to the citation count data, and generated a bubble map to visualize the results.^[14]^ The default parameters were used for the analyses and creation of bubble maps. The font size of the words in the bubble map indicates the frequency of occurrence (multiple appearances in a single publication counted as 1). Two words were close to each other if they co-occurred in the evaluated publications more frequently. Only keywords that appeared in at least 1.0% (n = 11) of the publications were analyzed and visualized. For the keyword map, a full counting method was used, meaning that each co-occurrence link carried the same weight. The default “association strength method” was used for the normalization of the co-occurrence matrix with default values of attraction and repulsion.

Approval from an ethics committee was not required since all data were recovered from the Web of Science database.

## 3 Results

**Figure 1:**
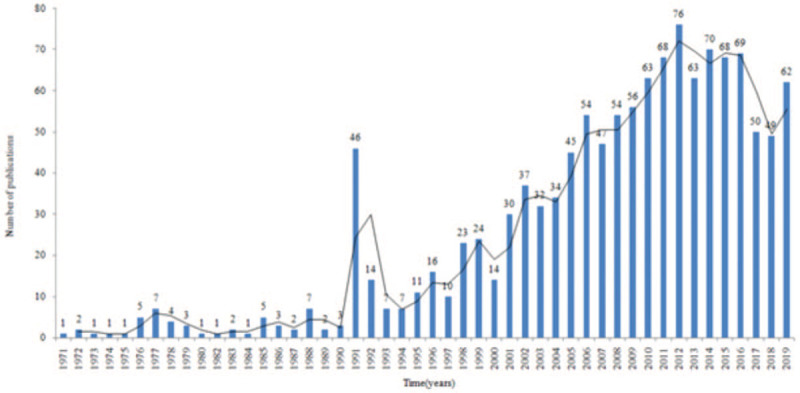
The number of publications on aristolochic acid nephropathy (AAN) every year.

We identified 1251 AAN-related records. The first article relating to AAN was published in 1971 by Prodanov and Astrug.^[15]^ Since then, the number of publications on AAN has gradually increased each year until 2000, after which there was an exponential growth in the past 2 decades (Fig. 1). The number of records during the last 5 years (2015–2019) accounted for 23.8% (298) of the total publications.

Based on the report of Zhou et al,^[3]^ the keywords “meeting abstract,” “letter,” “editorial material,” or “proceedings paper” were removed, leaving 923 original articles and 160 reviews that were enrolled for further analysis in this study; English (1047; 96.7%) was the primary language of the articles, followed by French (15; 1.4%) and German (7; 0.6%).

**Figure 2:**
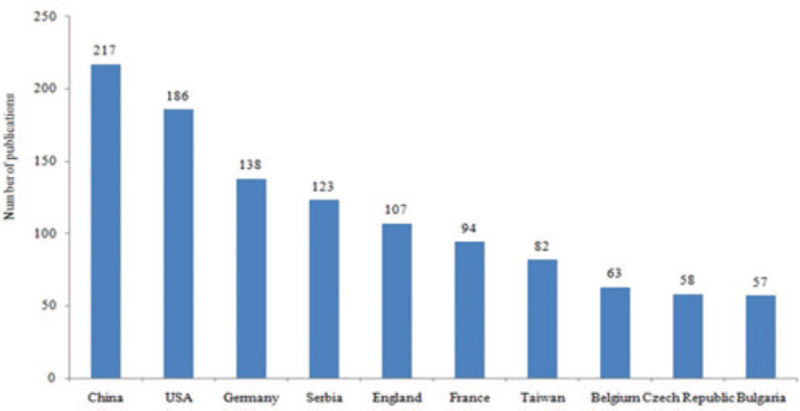
Top 10 most productive countries.

Authors from 79 different countries contributed to publications. Authors from China were the most numerous (217; 20.0%), followed by the USA (186; 17.2%) and Germany (138; 12.7%). China also had the highest number of publications. The 10 countries with the most number of publications are shown in Figure 2.

Table 1 shows the 10 journals with the most number of articles published on AAN, with *Kidney International* having the most number of articles (44; 4.1%), followed by *Food and Chemical Toxicology* (30; 2.8%), *Toxins* (24; 2.2%), *Nephrology Dialysis Transplantation* (23; 2.1%), *Renal Failure* (21; 1.9%), *Archives of Toxicology* (19; 1.8%), *Toxicology* (17; 1.6%), *PLOS One* (16; 1.5%), *American Journal of Kidney Diseases* (14; 1.3%), and *Chemical Research in Toxicology* (14; 1.3%).

The total number of citations for the publications was 39,970. Each publication was cited at least 36 times with an average of 36.91 citations per publication (ranging from 0 to 1769). The h-index of the1083 published articles and reviews was 91. The ranking of the 10 most cited publications associated with the use of AAN is shown in Table 2.

The 10 most cited articles had authors from 7 countries (India, Belgium, USA, England, Canada, Croatia, and France) and were published in 9 journals: *Lancet* (2), *New England Journal of Medicine* (1), *Molecular Nutrition & Food Research* (1), *Bulletin of the World Health Organization* (1), *Kidney International* (1), *Proceedings of the National Academy of Sciences of the United States of America* (1), *Mutagenesis* (1), *Journal of Animal Science* (1), and *International Journal of Coal Geology* (1).

**Figure 3:**
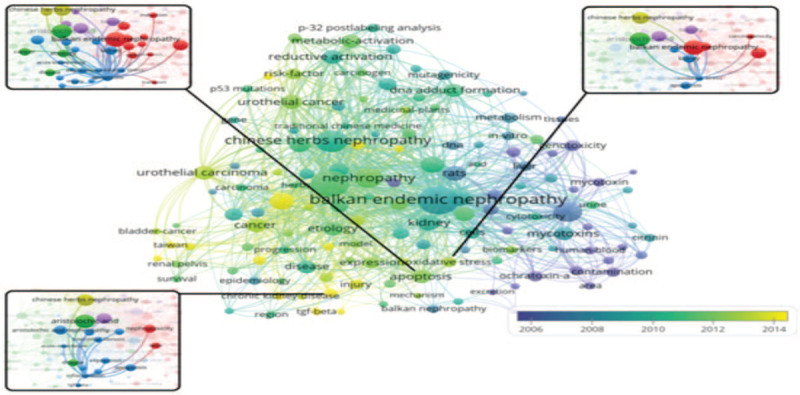
Bubble map visualizing words from keywords of the 1083 aristolochic acid nephropathy publications. Only words that appeared in at least 1.0% of the publications were analyzed and visualized.

There were 157 keywords that appeared in at least 1.0% (n = 11) of the evaluated publications. The top 20 recurring keywords are listed in Table 3. By analyzing the keywords of the 1083 publications, we found that the total link strength of keywords was related to the mechanism of AA toxicity, such as apoptosis (n = 73, citations per publication = 6.7%), inflammation (n = 58, citations per publication = 5.3%), and oxidative stress (n = 38, citations per publication = 3.5%). The bubble map of the total link strength of keywords, especially apoptosis, inflammation, and oxidative stress, is shown in Figure 3.

## 4 Discussion

For this scientometric study, we selected the Web of Science database as our source to retrieve all data regarding the research output in the field of AAN. The Web of Science database is the largest and one of the most reliable databases for publications and citations, providing quantitative (number of studies) as well as qualitative data, including impact (h-index).^[26]^

We included 1251 records regarding AAN in all languages. The number of writings has been growing continuously, with an exponential increase in the last 2 decades, demonstrating that AAN has been an interesting subject for scientific research in the field of nephropathy. China, the USA, and Germany were found to be the most productive countries in this regard.

The prevalence of AAN has increased worldwide over the last 2 decades, paralleling a soaring prevalence of apoptosis,^[27,28]^ oxidative stress,^[29,30]^ and inflammation,^[31,32]^ all of which are important mechanisms for AAN. Anti-inflammatory or anti-apoptotic agents could become potential treatment strategies in the future.^[27,33,34]^

Our study had several advantages and limitations. We used the Web of Science database as the source to retrieve all publications pertaining to AAN as it is the most recognized database for reviewing scientific literature in certain fields of research. This study is the first to analyze research activity in the field of AAN, showing an exponential increase in the number of publications. However, by using the Web of Science database alone, the contribution of other databases may have been underestimated. In addition, the results of the retrieval are biased to some extent, especially the highly cited article by Finkelman et al,^[25]^ which considered Balkan endemic nephropathy to be associated with the proximity of pliocene lignite deposits, conflicting with Markovic-Lipkovsk et al.^[9]^ Therefore, the precise cause of this phenomenon is a continuous process of constant exploration.

To our knowledge, this is the first bibliometric study to analyze and quantify global research productivity related to AAN. However, it should be mentioned that Zhang et al recently published a bibliometric analysis and systematic review of global publications regarding trends in AAs,^[3]^ whereas our study included only publications pertaining to AAN.

## 5 Conclusion

This study showed that apoptosis, oxidative stress, and inflammation are key topics in the mechanism of AAN and offer researchers and clinicians insights into this area of research.

## Author contributions

**Conceptualization:** Xiaohua Zhou.

**Data curation:** Hongjian Ji.

**Formal analysis:** Hongjian Ji.

**Funding acquisition:** Hongjian Ji.

**Investigation:** Jingyin Hu.

**Methodology:** Dean Guo.

**Project administration:** Jianxiang Song, Dean Guo.

**Software:** Hongjian Ji.

**Supervision:** Jianxiang Song.

**Validation:** Guozhe Zhang.

**Writing – original draft:** Hongjian Ji, Jingyin Hu, Guozhe Zhang.

**Writing – review & editing:** Xiaohua Zhou.
